# Development of an Immunoassay Method for the Sensitive Detection of Histamine and Tryptamine in Foods Based on a CuO@Au Nanoenzyme Label and Molecularly Imprinted Biomimetic Antibody

**DOI:** 10.3390/polym15010021

**Published:** 2022-12-21

**Authors:** Xinli Peng, Yongfeng Chen, Chunhui Gao, Yufeng Sun, Geoffrey I. N. Waterhouse, Zhixiang Xu

**Affiliations:** 1 College of Food Science and Engineering, Shandong Agricultural University, Tai’an 271018, China; 2 School of Chemical Sciences, The University of Auckland, Auckland 1142, New Zealand

**Keywords:** histamine, tryptamine, nanozyme, biomimetic immunoassay

## Abstract

In this paper, a novel biomimetic enzyme-linked immunoassay method (BELISA) was successfully established for the detection of histamine and tryptamine, based on catalytically active cupric oxide@gold nanoparticles (CuO@Au NPs) as a marker and a molecularly imprinted polymer (MIP) as the biomimetic antibody. Under optimized conditions, the detection limitations of the BELISA method for histamine and tryptamine were 0.04 mg L^−1^ and 0.14 mg L^−1^, respectively. For liquor spiked with histamine and tryptamine, the BELISA method delivered satisfactory recoveries ranging from 89.90% to 115.00%. Furthermore, the levels of histamine and tryptamine in fish, soy sauce, and rice vinegar samples were detected by the BELISA method and a high performance liquid chromatography method, with no significant difference between the two methods being found. Although the catalytic activity of nanozymes is still lower than that of natural enzymes, the BELISA method could still sensitively determine the histamine and tryptamine levels in food samples.

## 1. Introduction

Histamine and tryptamine, as the main biogenic amines, are formed by the decarboxylation of amino acids during food processing and storage [1]. Some studies have shown that the excessive intake of histamine and tryptamine may lead to hypotension, headaches, skin flushing, and edema [2,3]. The level of histamine and tryptamine in foods is, therefore, considered an important index to evaluate food safety [4]. Accordingly, establishing a sensitive method for the detection of histamine and tryptamine in foods is extremely important for human health.

In recent years, various analytical methods for the detection of histamine and tryptamine have been reported, especially high-performance liquid chromatography (HPLC) and gas chromatography (GC) [5,6]. HPLC possesses good accuracy, but a complex derivatization procedure using dansyl chloride is required before analysis [7]. GC has a lower limit of detection (LOD), but similar to HPLC, it requires expensive equipment [8]. These limitations have prompted the progress of enzyme-linked immunosorbent assays (ELISA) for the sensitive and rapid detection of histamine and tryptamine [9,10]. However, the application of the ELISA method is presently hampered by the poor stability of natural antibodies [11]. This motivates the search for biomimetic antibodies with high stability to replace natural antibodies in ELISA.

Molecularly imprinted polymers (MIPs) are increasingly being used in analytical chemistry due to their ability to selectively recognize specific analytes. Their good stability and strong specific recognition ability suggest the potential for their use as biomimetic antibodies in ELISA [12,13]. In recent years, several studies using MIPs in biomimetic enzyme-linked immunoassays (BELISA) have been reported [14,15]. However, most BELISA methods use natural enzymes as markers. Natural enzymes are large bulky macromolecules and thus introduce shielding effects, accordingly interfering with marker recognition by the MIPs [14]. Therefore, it is necessary to find new markers with simple structures to replace natural enzymes in BELISA [16].

Nanozymes are nanomaterials that have enzyme-like characteristics [17]. For example, cupric oxide nanoparticles (CuO NPs) with haloperoxidase (HPO) activity have been reported, and thus applied, in sensors and biomedical fields [18,19]. Wang and co-works explored the HPO activity of CuO NPs and established a novel assay method for glucose detection [20]. However, the application of CuO NPs in ELISA has been limited due to their poor catalytic activity [21]. Gold nanoparticles (Au NPs) possess unique optical properties and peroxidase-like activity, leading to their application in biosensors and surface-enhanced Raman spectroscopy [22]. Combining CuO NPs with HPO-like activity and Au NPs with peroxidase-like activity is expected to deliver novel nanozymes for BELISA.

In this paper, CuO@Au NPs with peroxidase activity were prepared through the decoration of CuO with Au NPs. A hydrophilic MIP for histamine and tryptamine was then polymerized directly into the well surface of a 96-well plate by bulk polymerization. Using CuO@Au NPs as the marker and the MIP film as a biomimetic antibody, a novel BELISA method to detect the histamine and tryptamine was developed. The factors affecting the determination of each analyte using the method (such as the concentrations of H_2_O_2_, TMB, and nanozyme conjugate and the dilution of the standard sample solution) were all optimized, allowing the properties of the BELISA method (sensitivity, selectivity, repeatability, accuracy, and applicability) to be evaluated.

## 2. Results and Discussions

### 2.1. Characterization of the MIP

**Figure 1:**
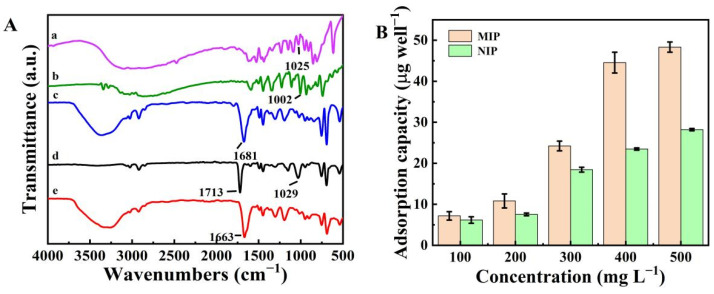

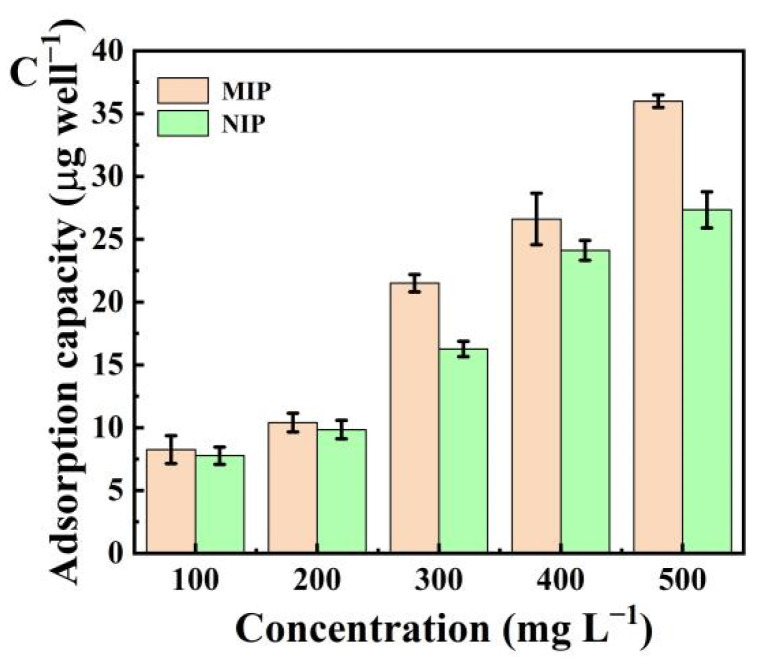
(**A**) FT-IR spectra for (a) histamine dihydrochloride, (b) tryptamine, (c) NIP, (d) MIP before elution, and (e) MIP after elution; (**B**) Adsorption isotherms for histamine on the MIP and NIP; (**C**) Adsorption isotherms for tryptamine on the MIP and NIP.

Figure 1A shows FT-IR spectra for the template molecules (histamine dihydrochloride and tryptamine), NIP, and MIP. In the FT-IR spectra of the MIP and NIP, the absorption peaks at 1681, 1713, and 1663 cm^−1^ corresponded with the C=O stretching vibrations of MAA [23]. The histamine dihydrochloride, tryptamine, and the MIP before elution showed absorption peaks at 1025, 1022, and 1029 cm^−1^ that were assigned to the C-N stretching vibrations of histamine and tryptamine. The small difference in the C-N absorption peak position in the MIP (before elution) was likely due to the amino of template molecules and the carbonyl of MAA forming hydrogen bonds [16]. After the elution step, the C-N peak disappeared, indicating the template molecules (histamine dihydrochloride and tryptamine) had been successfully eluted from the MIP.

Next, the adsorption ability of the MIP was measured (Figure 1B,C). The adsorption capacities of the MIP were higher than that of the NIP at the same concentration. When the concentration of histamine and tryptamine was 500 mg L^−1^, the imprinting factors (IF = K_M_/K_N_, where K_M_ and K_N_ are the adsorption amount of the template molecule by MIP and NIP, respectively) of the histamine and tryptamine were calculated to be 1.71 and 1.32, respectively. Results demonstrated that the MIP was more useful for the development of the biomimetic antibody immunoassay.

### 2.2. Characterization of CuO@Au NPs

The Fourier-transform infrared spectrum of CuO NPs (Figure S1A) showed absorption peaks at 608 and 495 cm^−1^ which were assigned to Cu-O stretching vibrations in cupric oxide, confirming the successful preparation of CuO NPs [24]. After the decoration of the CuO NPs with Au NPs, and new ultraviolet-visible (UV-Vis) peak appeared at 545 nm (Figure S2B), corresponding to the surface plasmon resonance of Au NPs [25]. The data confirmed that CuO@Au NPs were successfully prepared.

### 2.3. Evaluation of the Catalytic Ability of the CuO@Au NPs

**Figure 2:**
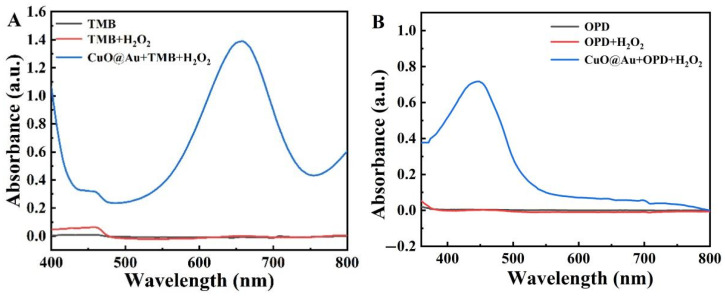

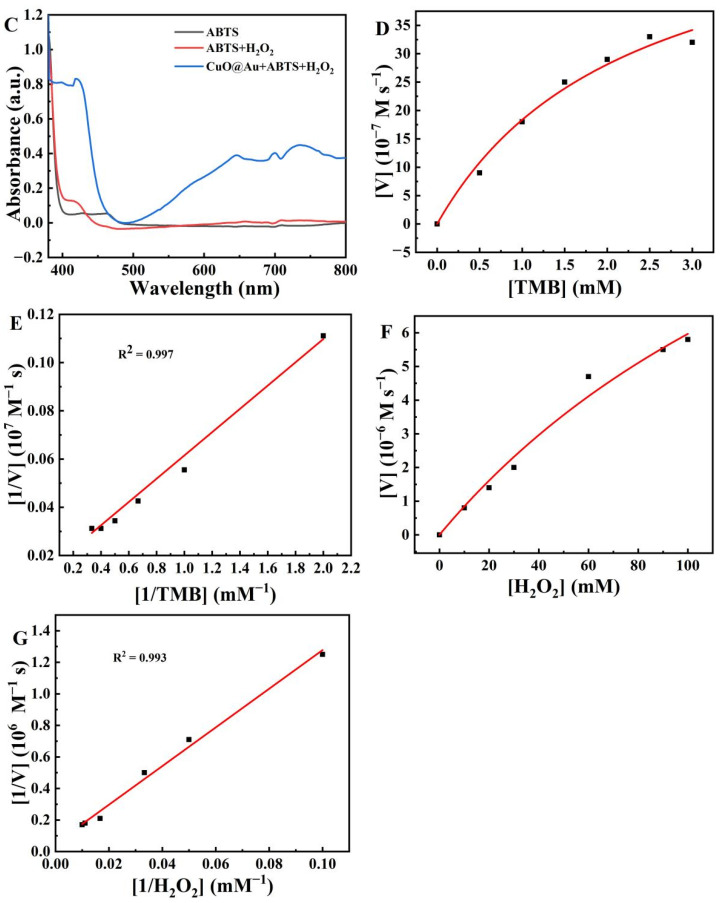
UV-Vis spectra for the oxidation of different substrates by the CuO@Au nanozyme in the presence of H_2_O_2_ (**A**) with TMB as substrate at a fixed H_2_O_2_ concentration; (**B**) with OPD as substrate at a fixed H_2_O_2_ concentration; (**C**) with ABTS as substrate at a fixed H_2_O_2_ concentration; (**D**) Michaelis–Menten curve for TMB oxidation by CuO@Au NPs in the presence of H_2_O_2_ at different TMB concentrations; (**E**) Corresponding Lineweaver–Burk plot for data in (**D**); (**F**) Michaelis–Menten curve for TMB oxidation by CuO@Au NPs in the presence of TMB at different H_2_O_2_ concentrations; (**G**) Corresponding Lineweaver–Burk plot for the data in (**F**).

The catalytic ability of CuO@Au NPs was next investigated using TMB, OPD, and ABTS as substrates. Figure 2A–C shows that all three substrates were oxidized by CuO@Au NPs, with the oxidation of each substrate giving rise to absorbance maxima at 652 nm, 450 nm, and 417 nm, respectively. The highest absorbance was found when TMB was used as a substrate. These results demonstrate that CuO@Au NPs possessed peroxidase-like activity, with TMB being the most suitable chromogenic substrate.

Next, the effect of pH (in PBST buffer) on the catalytic ability of CuO@Au NPs was explored (Figure S2A). Experiments were performed over the pH range of 2.5–6.0. The maximum absorbance was obtained at a pH level of 4.0. Accordingly, the subsequent experiments were carried out at a pH level of 4.0.

To test the thermal stability of the CuO@Au NPs, the catalyst was incubated at 30–100 °C with TMB and H_2_O_2_ for 10 min. CuO@Au NPs exhibited the highest catalytic activity when incubated at 40 °C (Figure S2B), with 77% of this activity still being retained after incubation at 100 °C. Results confirmed that CuO@Au NPs possessed good thermal stability. Moreover, the storage stability of CuO@Au NPs was investigated at room temperature for 7 days (Figure S2C). The CuO@Au NPs retained 98% of their initial activity after 7 days, thus showing a negligible change in catalytic activity during storage (as expected for an inorganic-based catalyst).

The concentration of Cl^−^ was found to regulate the catalytic ability of CuO@Au NPs. In the presence of Cu ions, H_2_O_2_ can catalyze the oxidation of Cl^−^ to form reactive species, thus promoting the oxidation of the substrate [20]. Results showed that the catalytic ability of CuO@Au NPs increased with the concentration of Cl^−^ up to 80 mmol L^−1^ (Figure S2D), and decreased slightly at higher Cl^−^ concentrations. Therefore, 20 μL of an 80 mmol L^−1^ chloride solution was used for the next experiments.

Next, the steady-state kinetics of the TMB oxidation reaction by CuO@Au NPs was studied. When TMB and H_2_O_2_ were used as substrates, the catalytic activity of CuO@Au NPs conformed to Michaelis–Menten kinetics (Figure 2D,F). The corresponding kinetic parameters (K_m_, Michaelis–Menten constant; V_max_, maximum reaction velocity) of the CuO@Au NPs were calculated using Lineweaver–Burk plots (Figure 2E,G, respectively). With TMB as the substrate (at a fixed concentration of H_2_O_2_), the calculated K_m_ and V_max_ values were 21.56 mM and 17.89 × 10^−6^ M s^−1^, respectively. With H_2_O_2_ as the substrate (at a fixed concentration of TMB), the K_m_ and V_max_ values were 236.46 mM and 19.30 × 10^−6^ M s^−1^, respectively. The K_m_ values for CuO@Au NPs were larger than that reported for HRP (Table S1), confirming that CuO@Au NPs possessed a lower affinity for these substrates compared to HRP. However, the V_max_ values for CuO@Au NPs were larger than those of HRP, indicating that the H_2_O_2_ decomposition and subsequent TMB oxidation over CuO@Au NPs were faster than on HRP.

### 2.4. Optimization of BELISA Conditions

The concentrations of H_2_O_2_ and TMB were optimized to find the optimal reaction conditions (Figure S3A,B). The maximum absorbance at 652 nm was obtained when the concentrations of H_2_O_2_ and TMB were 6 mol L^−1^ and 3 mmol L^−1^, respectively. Accordingly, these concentrations of H_2_O_2_ and TMB were used in the subsequent experiments.

We also optimized the concentration of the nanozyme conjugates to enhance the sensitivity of the BELISA method. Figure S3C,D shows the effect of the nanozyme conjugate dilution ratio (1:2, 1:4, 1:8, 1:16, 1:32, 1:64). When the nanozyme conjugate dilution ratio was 1:4, the absorbance values were in the range of 0.8–1.0, which was near-ideal for colorimetric determinations. Therefore, a dilution ratio of 1:4 was selected for subsequent experiments.

To further increase the sensitivity of the BELISA method, the solvent used to prepare the standard solutions was investigated. As shown in Figure S3E,F, the BELISA method possessed the highest sensitivity for the detection of histamine and tryptamine when methanol was used as the solvent. Hence, methanol was used as the solvent to prepare the standard solutions and sample extracts for the BELISA analysis.

### 2.5. Standard Curve of the BELISA Method

**Figure 3:**
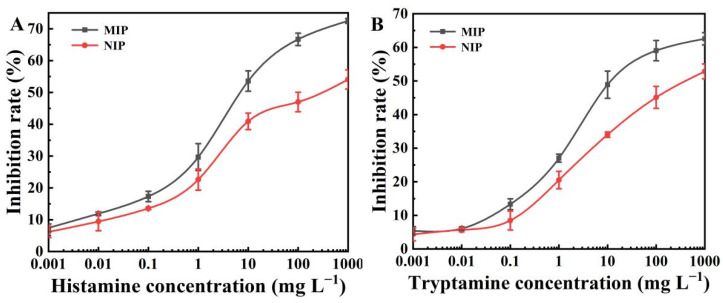
(**A**) BELISA standard curves for histamine using NIP and MIP as the artificial antibody; (**B**) BELISA standard curves for tryptamine using NIP and MIP as the artificial antibody.

In this paper, a novel BELISA method was presented. A competitive reaction occurred, wherein the immunoprobe and template molecules competed to bond to the recognition sites of the MIP. Figure 3 shows the standard curves of histamine and tryptamine at concentrations between 0.001 and 1000 mg L^−1^. Under the optimum testing conditions, the LOD (IC_15_) and sensitivity (IC_50_) values of the BELISA method were 0.04 and 6.80 mg L^−1^ for the histamine, and 0.14 and 12.02 mg L^−1^ for the tryptamine, respectively.

### 2.6. Selectivity of the BELISA Method

Standard curves with the MIP and NIP as artificial antibodies were established to investigate the selectivity of the BELISA method (Figure 3). When the concentration of histamine and tryptamine was 10 mg L^−1^, inhibition rates of 53.59% and 48.90% were found for the MIP, respectively, with these values being higher than those obtained with the NIP (40.92% and 34.04%, respectively).

**Figure 4:**
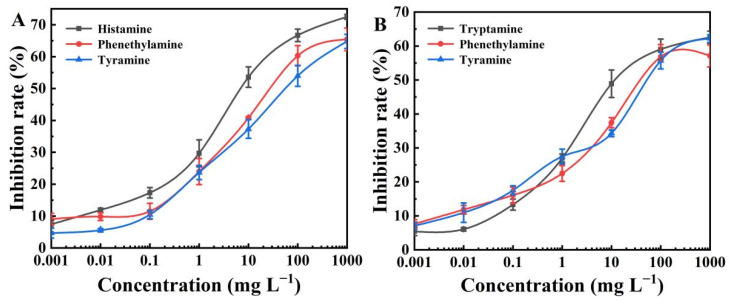
(**A**) BELISA standard curves for histamine, phenethylamine, and tyramine; (**B**) BELISA standard curves for tryptamine, phenethylamine, and tyramine.

Cross-reactivity (CR) was also measured to verify the specificity of the BELISA method. Phenethylamine and tyramine, as the structural analogs of histamine and tryptamine, were used to perform a competitive assay (Figure 4). Results in Tables S2 and S3 show that the CR values of histamine with phenethylamine and tyramine were 22.32% and 11.35%, respectively. The CR values of tryptamine with phenethylamine and tyramine were 25.86% and 20.31%, respectively. These results confirmed that the BELISA method possessed a much higher selectivity toward histamine and tryptamine compared to their structurally related compounds (as expected, since the BELISA method used a histamine and tryptamine-imprinted polymer).

### 2.7. Accuracy and Applicability of the BELISA Method

The recovery of histamine and tryptamine from spiked liquor samples was used to investigate the accuracy of the method. In Table 1, histamine and tryptamine recoveries using the BELISA method were in the range of 89.90–115.00%, with RSDs of 1.28–5.35%, indicating that the BELISA method possessed excellent accuracy.

The contents of histamine and tryptamine in food (fish, soy sauce, and rice vinegar) were analyzed by BELISA and HPLC to evaluate the applicability of the method. Table S4 shows that no significant differences between the two methods were obtained (*p* > 0.05), thus confirming the excellent applicability of the BELISA method.

### 2.8. Advantages and Disadvantages of the BELISA Method

Table S5 compared the BELISA method with previously reported methods [26,27,28,29]. The LOD of the BELISA method was lower than most previous methods. Furthermore, the use of a MIP as a biomimetic antibody provided better stability and reliability than a natural antibody. More importantly, the MIP-filled 96-well plate could be reused. The inhibition rate of histamine showed no obvious change after three uses (Figure S4). The inhibition rate was reduced by less than 15% after five uses. Accordingly, the cost of the analytical method was substantially reduced by being able to reuse the 96-well plate. Further, the CuO@Au NPs nanozyme was smaller than natural enzymes, such as HRP. Hence, when CuO@Au NPs were employed as the marker, the ability of the MIP to recognize nanozyme conjugates was improved. However, the catalytic ability of the nanozyme was lower than that of HRP, and the recognition ability of the imprinted membrane was also inferior to that of the biological antibody. Nonetheless, CuO@Au NPs represented an effective peroxidase mimic.

## 3. Material and Methods

### 3.1. Materials, Chemicals, and Apparatus

The fish, soy sauce, rice vinegar, and liquor samples were bought from a supermarket (Tai’an, China). The source of the chemicals used in the research are listed in the supplementary information.

Fourier-transform infrared (FT-IR) spectra were measured on an infrared spectrometer (Nicolet iS 10, Thermo, Waltham, MA, USA). The UV-Vis absorbances detected on a Plate Reader (SpectraMax M5, Sunnyvale, CA, USA). HPLC (SPD-20A, Shimadzu, Kyoto, Japan) were used to detect histamine and tryptamine in the fish, soy sauce, rice vinegar, and liquor samples. The HPLC procedure is described in the supplementary information.

### 3.2. Synthesis of CuO@Au NPs

The CuO NPs were synthesized according to a previously reported method [20]. Then, 0.5 mL of glacial acetic acid and 150 mL of a copper acetate monohydrate solution (0.02 mol L^−1^) were mixed in a 300 mL flask with the reflow device and then heated to boiling under constant stirring. Next, 0.6 g of NaOH was added to the copper acetate solution, resulting in the formation of a black precipitate. After the mixture had cooled, the product was collected by centrifugation at 2664 × *g* for 10 min. The black CuO NPs powder was dried under a vacuum at 45 °C after washing with DDW once and ethanol three times.

The CuO@Au NPs were prepared using a simple citrate reduction method. Briefly, 40 mg of CuO NPs and 20 mL of an aqueous HAuCl_4_ solution (1 mmol L^−1^) were transferred to a beaker, and the resulting dispersion was heated to boiling under constant stirring. After quickly adding 5 mL of a sodium citrate solution (0.39 mmol L^−1^), the resulting dispersion was stirred vigorously for 10 min. The CuO@Au NPs were collected by centrifugation, then washed three times with DDW and dried under a vacuum at 45 °C.

### 3.3. Preparation of Nanozyme Conjugates

**Scheme 1:**
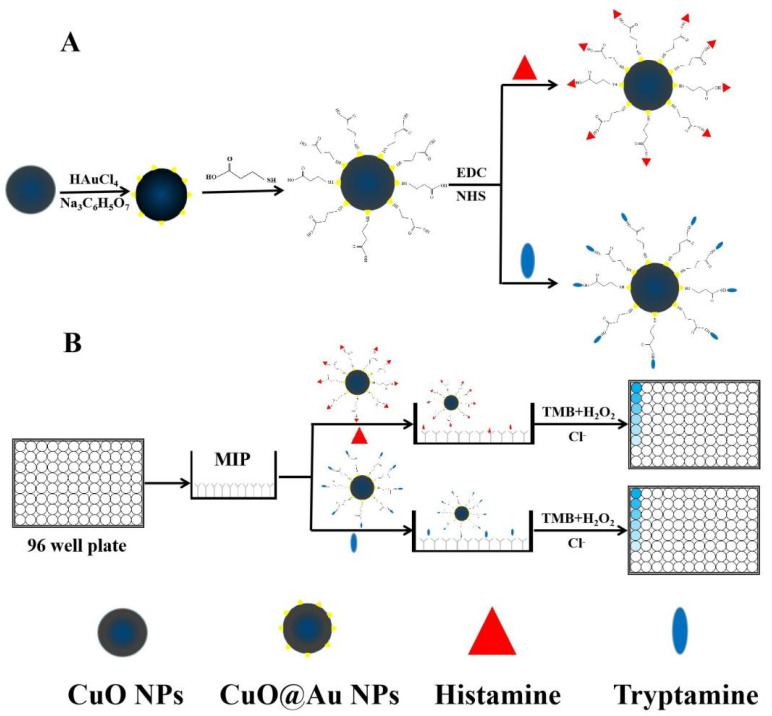
(**A**) Schematic illustration of the synthesis of CuO@Au NPs; (**B**) Schematic illustration of histamine and tryptamine detection using the BELISA method.

First, 5.0 mL of an aqueous CuO@Au NP dispersion (0.5 mg mL^−1^) and 1.5 mL of mercaptopropanoic acid (0.01 mol L^−1^) were transferred to a round-bottom flask, and the resulting mixture was stirred for 12 h. The resulting dispersion was centrifuged at 5994× *g* for 10 min, after which, the solid was redispersed in 5.0 mL of DDW. After adding 1.5 mL of EDC (0.01 mol L^−1^) and 1.5 mL of NHS (0.01 mol L^−1^), the resulting dispersion was stirred for 4 h at room temperature. Subsequently, 1.5 mL of histamine (0.01 mol L^−1^) was injected, and the resulting mixture was stirred for 4 h. The dispersion was centrifuged at 5994× *g* for 10 min, after which, the obtained histamine nanozyme conjugate was dispersed in 5.0 mL of PBST. A tryptamine nanozyme conjugate was synthesized according to the same general procedure. The syntheses of the nanoenzyme conjugates are shown in Scheme 1A.

### 3.4. Preparation of the MIP Film on the Surface of the 96-Well Plate

The MIP films were prepared on the surface of a 96-well plate. Firstly, 92.04 mg of histamine dihydrochloride and 80.11 mg of tryptamine were dissolved in 20 mL of DMSO. After MAA (3.0 mmol) was transferred to the above solution and the mixture was magnetically stirred for 30 min at room temperature, AIBN (40 mg) and EGDMA (8.0 mmol) were added and the resulting mixture was continuously stirred for 1 h. After ultrasonication for 2 min, 200 μL of the above solution was set in all wells, and the 96-well plates were sealed and incubated in a bag with N_2_ at 45 °C for 16 h. After the completion of the MIP polymerization, the 96-well plate was cleaned using DDW and methanol to wipe off any residual monomers. Next, the plate was ultrasonically washed with methanol/acetic acid (9:1, *v*/*v*) and methanol for 8 h and 4 h, respectively. Finally, the MIP-filled 96-well plates were dried at room temperature.

Furthermore, a non-imprinted membrane (NIP) was prepared by the same process, but without the addition of template molecules (histamine dihydrochloride and tryptamine).

### 3.5. BELISA Procedure

The BELISA procedure is described in Scheme 1B. The 96-well plates were washed three times with PBST, after which, 100 μL of a methanolic histamine standard solution or sample extract was transferred to specific wells. Methanol was added to the control (100 μL) and blank wells (200 μL). Meanwhile, 100 μL of nanozyme conjugates solution (in PBST buffer) was placed in all wells except for the blank wells. After the plates were continuously shaken for 1 h and cleaned with PBST five times, 20 μL of a NaCl solution (80 mmol L^−1^) and 200 μL of a substrate solution (100 μL TMB and 100 μL H_2_O_2_) were added to all wells. Then, the wells were incubated for 10 min. Subsequently, the chromogenic reactions were interrupted by joining 50 μL of H_2_SO_4_. The absorbances at 450 nm, corresponding to the oxidation product of TMB, were recorded, and the inhibition rates were calculated.

### 3.6. Sample Preparation

To prove accuracy, the liquor samples spiked with histamine and tryptamine were determined by the BELISA method. The samples were prepared as follows: 1.0 mL of liquor and 1.0 mL of a histamine and tryptamine standard solution (2.0 mg L^−1^, 5.0 mg L^−1,^ or 10.0 mg L^−1^) were transferred to a 15 mL test tube and derivatized using dansyl chloride. Next, 5.0 mL of diethyl ether and 0.5 g of NaCl were joined, and the resulting mixture was then vortexed for 2 min. Then, the mixture was left to stand until phase separation, the organic layer was placed into a tube and evaporated. The solution was filtered by a 0.22 μm film after the residue was redissolved in methanol (1.0 mL). Finally, the concentration of histamine and tryptamine was determined by BELISA.

Liquid sample (soy sauce and rice vinegar) treatments are described in the supplementary information.

### 3.7. Statistical Analysis of Data

The differences between the results were analyzed using an ANOVA test.

## 4. Conclusions

The high-sensitivity BELISA method for rapidly detecting histamine and tryptamine in foods was successfully developed using CuO@Au NPs as a marker and a MIP as a biomimetic antibody. The BELISA method exhibited good sensitivity, repeatability, selectivity, and accuracy. Under optimized testing conditions, the IC_15_ and IC_50_ were 0.04 and 6.80 mg L^−1^ for the histamine, and 0.14 and 12.02 mg L^−1^ for the tryptamine, respectively. This work provides an extremely effective and low-cost method for the quantification of histamine and tryptamine.

## Figures and Tables

**Table 1:** Recovery tests for histamine and tryptamine in spiked liquor samples using the BELISA (n = 3).

Sample	Original Level (mg L^−1^)		Added Level (mg L^−1^)	Found Level (mg L^−1^ ±SD)		Recovery (%, ±RSD)	
	Histamine	Tryptamine		Histamine	Tryptamine	Histamine	Tryptamine
**Liquor**	1.64	1.72	2	3.76 ± 0.07	4.02 ± 0.16	106.00 ± 3.51	115.00 ± 5.35
			5	6.50 ± 0.22	7.26 ± 0.20	97.20 ± 4.58	110.80 ± 3.55
			10	10.63 ± 0.29	11.56 ± 0.13	89.90 ± 3.20	98.40 ± 1.28

## Data Availability

The data presented in this study are available on request from the corresponding author.

## Supplementary Materials

The following supporting information can be downloaded at: https://www.mdpi.com/article/10.3390/polym15010021/s1. Figure S1: (A) FT-IR spectrum of CuO NPs; (B) UV-Vis spectra of CuO NPs and CuO@Au NPs; Figure S2: Effect of different parameters on TMB oxidation by CuO@Au NPs in the presence of H_2_O_2_. TMB oxidation gave rise to an absorbance maximum at 652 nm: (A) Effect of the pH; (B) Effect of reaction temperature; (C) Effect of the storage time; and (D) Effect of Cl^-^; Figure S3: Effect of substrate concentration and solvent on TMB oxidation by CuO@Au NPs in the presence of H_2_O_2_. TMB oxidation gave rise to an absorbance maximum at 652 nm: (A) Effect of the H_2_O_2_ concentration; (B) Effect of the TMB; (C) Effect of the dilution ratio of the histamine immunoprobe; (D) Effect of the dilution ratio of tryptamine immunoprobe; (E) Effect of the standard solution solvent on the inhibition rate of histamine; (F) Effect of the standard solution solvent on the inhibition rate of tryptamine; Figure S4: Resuability of the MIP-filled 96-well plate; Table S1: Comparison of the Michaelis-Menten constant (K_m_) and maximum reaction rate (V_max_) for different catalysts; Table S2: Chemical structures, IC50 and cross-reactivity of histamine and its structural analogues; Table S3: Chemical structures, IC50 and cross-reactivity of tryptamine and its structural analogues; Table S4: Comparison of the BELISA method and HPLC method for the detection of histamine and tryptamine in different foods (n = 3); Table S5: Comparison of the BELISA method with other methods for histamine and tryptamine detection. Reference [30] is cited in the Supplementary Materials.
