# Supplementary material for: Development of an Immunoassay Method for the Sensitive Detection of Histamine and Tryptamine in Foods Based on a CuO@Au Nanoenzyme Label and Molecularly Imprinted Biomimetic Antibody
Source: Polymers (Basel). 2022 Dec 21;15(1):21. doi: 10.3390/polym15010021 (PMC9823797; doi:10.3390/polym15010021)
Supplement: Supplementary file 1 [file polymers-15-00021-s001.zip › polymers-2043040-SM.pdf]

Supplementary Materials

Development of an Immunoassay Method for the Sensitive Detection of Histamine and Tryptamine in Foods Based on a CuO@Au Nanoenzyme Label and Molecularly Imprinted Biomimetic Antibody

Xinli Peng [1], Yongfeng Chen [1], Chunhui Gao [1], Yufeng Sun [1], Geoffrey I. N. Waterhouse [2] and Zhixiang Xu [1,*]

[1] College of Food Science and Engineering, Shandong Agricultural University, Tai'an 271018, China; xinlipeng1103@163.com (X.P.); chenyongfeng0708@163.com (Y.C.); haigangvz129@163.com (C.G.); sunyufeng1208@163.com (Y.S.)

[2] School of Chemical Sciences, The University of Auckland, Auckland 1142, New Zealand; g.waterhouse@auckland.ac.nz

* Correspondence: zhixiangxu@sina.com; Tel.: +86-538-824-2263; Fax: +86-538-824-2482

Materials and Methods

Materials, chemicals and apparatus

2,2-azobisisobutyronitrile (AIBN), methacrylic acid (MAA), acetic acid and dimethyl sulfoxide (DMSO) were obtained by Tianjin Kaitong Chemical Reagent Co., Ltd. (Tianjin, China). 1-(3-Dimethylaminopropyl)-3-ethylcarbodiimine hydrochloride (EDC), N-hydroxysuccinimide (NHS) and 3-mercaptopropionic acid were obtained from Macklin Biochemical Technology Co., Ltd. (Shanghai, China). The acetonitrile and benzoyl chloride were chromatographic grade. All other chemicals and reagents were analytical grade. Double-distilled water (DDW, 18.20 MΩ cm^{-1}) was obtained from an Aike ultrapure water instrument (Chengdu, China).

The conditions of HPLC were descried below: A mixture of ammonium acetate (0.01 mol L^{-1}) and acetonitrile (10:90, v/v) was used as mobile phase A. A mixture of ammonium acetate (0.01 mol L^{-1}) and acetonitrile (90:10, v/v) was used as mobile phase B. HPLC was used a gradient elution method (0 min: 60 % A; 22 min: 85 % A; 25 min: 100 % A; 32 min: 100 % A; 32.01 min: 60 % A; 37 min: 60 % A). The column temperature was 35 °C and the injection volume was 20 μL. The flow rate was 0.8 mL min^{-1} , with the detection wavelength set at 254 nm.

Sample preparation

The liquid samples (soy sauce and rice vinegar) were prepared as described above for the histamine and tryptamine standard solution. The fish sample was prepared as follows: 10 g of fish was placed in a 100 mL conical flask. Next, 20 mL of 5% trichloroacetic acid solution was added, after which the mixture was shaken for 30 min and then centrifuged at 4995×g for 10 min. The supernatant was transferred to a 50 mL volumetric flask and diluted to 50 mL with 5% trichloroacetic acid solution. Subsequently, 10 mL of the extract solution and 0.5 g of NaCl were transferred to a 25 mL test tube. The mixture was then vortexed for 5 min and the organic phase formed collected. Next, 5 mL of a n-butanol/chloroform (1:1) mixture was added and the mixture vortexed for 5 min. The mixture was the centrifuged at 4995×g for 5 min, and the upper aqueous phase transferred to another 10 mL centrifuge tube. Next, 200 μL of HCl (1 mol L^{-1}) was added to 5 mL of the aqueous extract, after which the mixture was evaporated to dryness with N_2 in a water bath at 40 °C. The residue was then dissolved with 1 mL of HCl (0.1 mol L^{-1}), after which the samples was derivatized as described above for the liquor samples. Finally, the extract solution was analyzed by HPLC and the BELISA method.

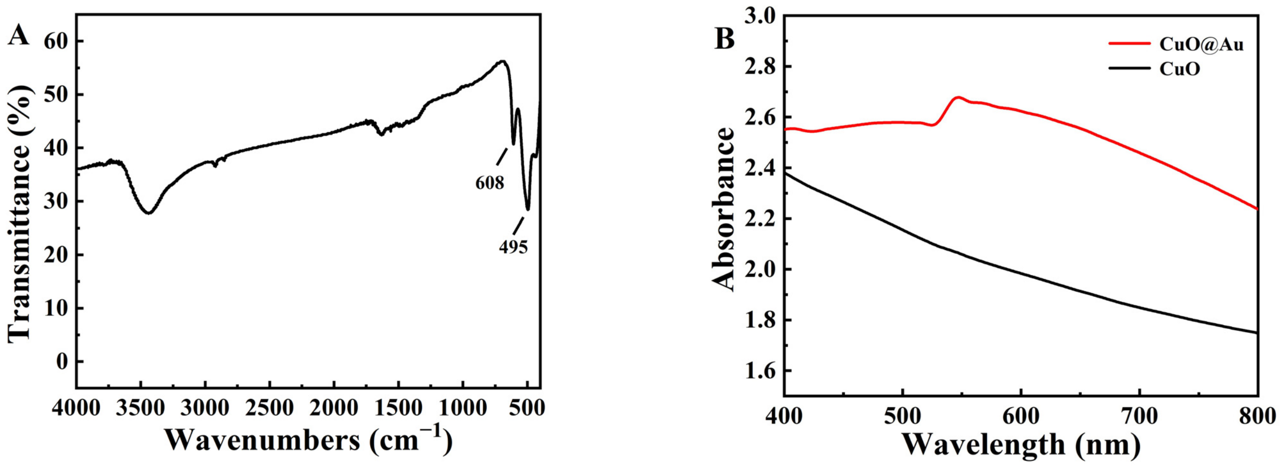

Figure S1. (A) FT-IR spectrum of CuO NPs; (B) UV-Vis spectra of CuO NPs and CuO@Au NPs.

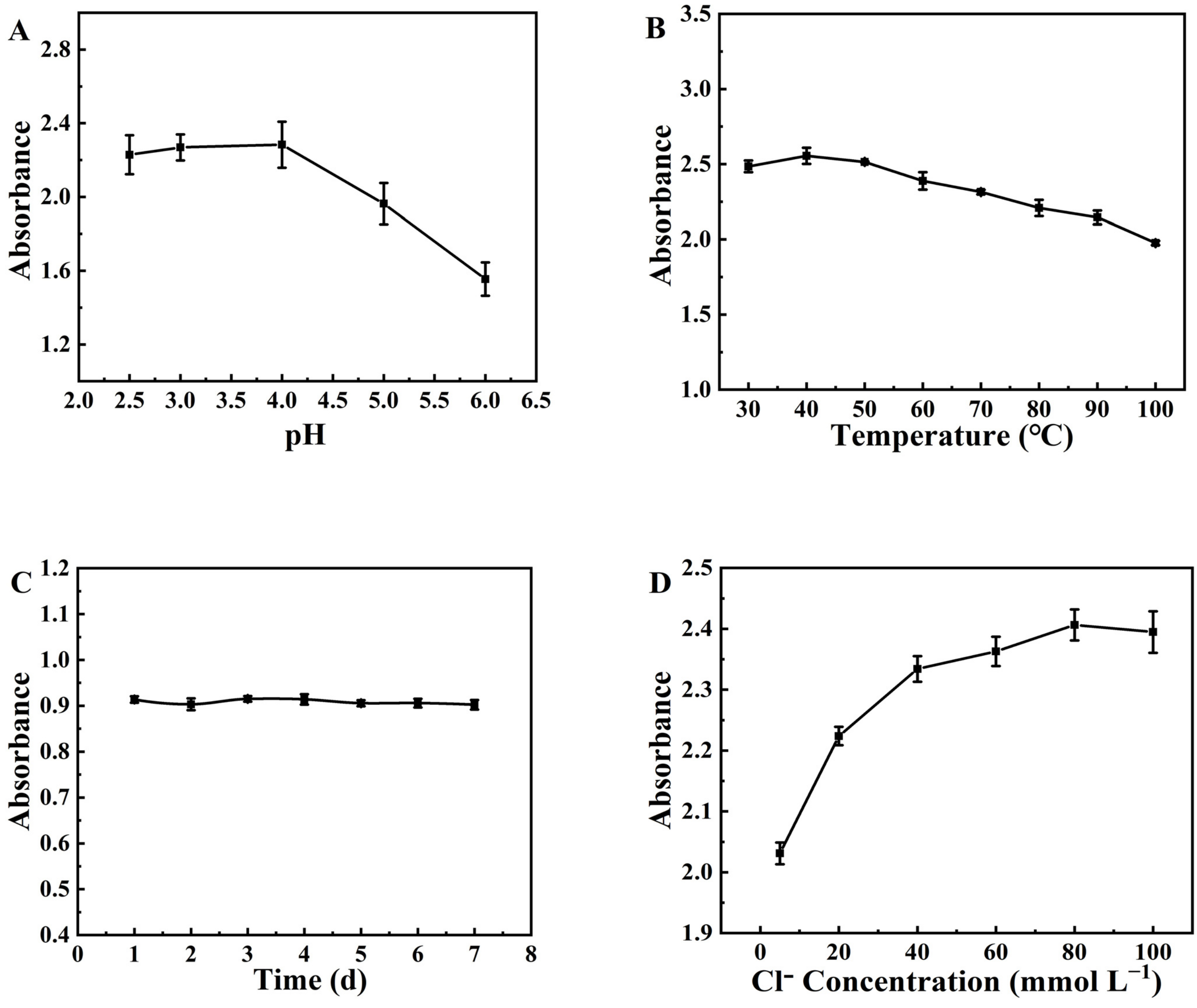

Figure S2. Effect of different parameters on TMB oxidation by CuO@Au NPs in the presence of H_2O_2. TMB oxidation gave rise to an absorbance maximum at 652 nm: (A) Effect of the pH; (B) Effect of reaction temperature; (C) Effect of the storage time; and (D) Effect of Cl^-.

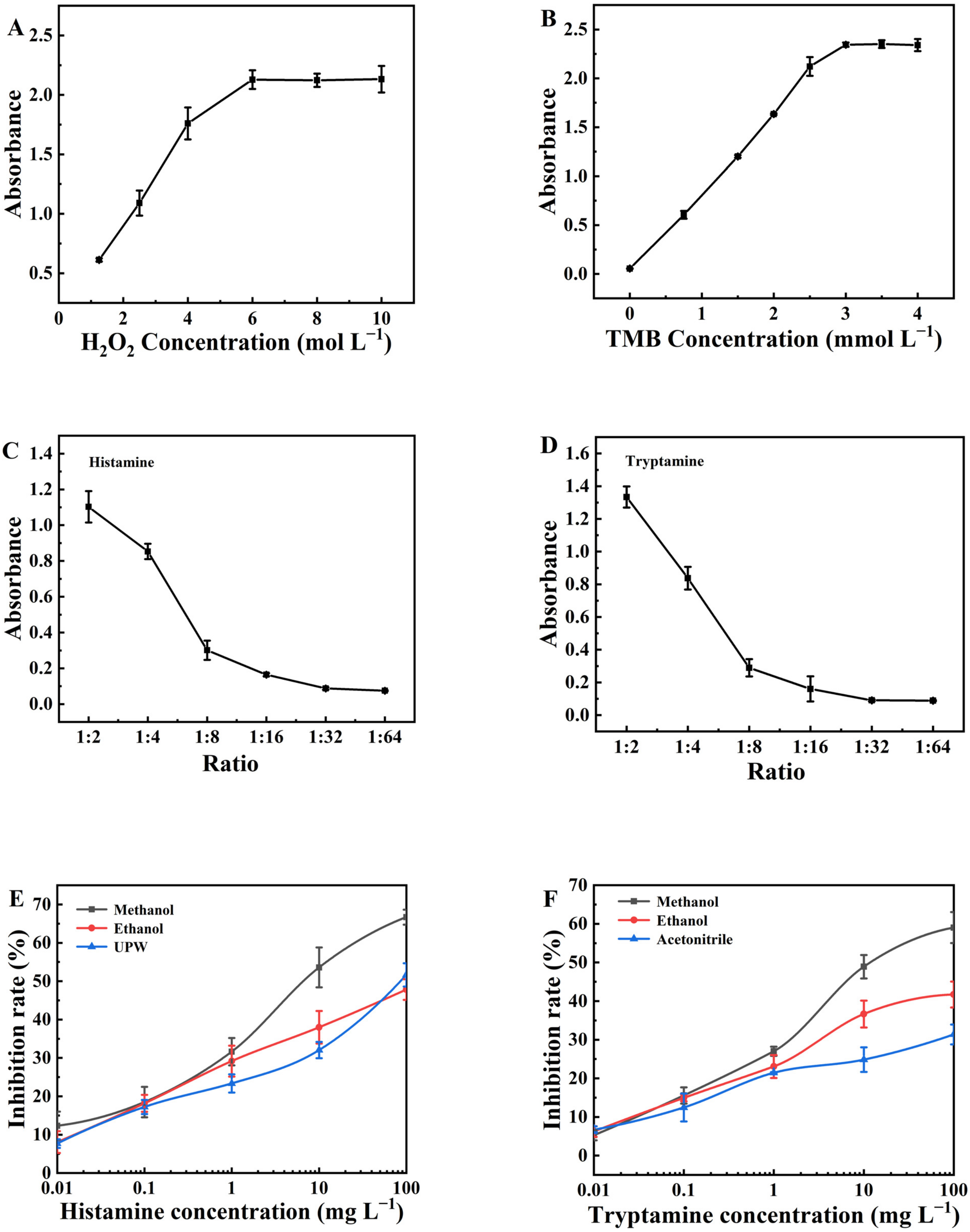

Figure S3. Effect of substrate concentration and solvent on TMB oxidation by CuO@Au NPs in the presence of H_2O_2. TMB oxidation gave rise to an absorbance maximum at 652 nm: (A) Effect of the H_2O_2 concentration; (B) Effect of the TMB; (C) Effect of the dilution ratio of the histamine im-

munoprobe; (D) Effect of the dilution ratio of tryptamine immunoprobe; (E) Effect of the standard solution solvent on the inhibition rate of histamine; (F) Effect of the standard solution solvent on the inhibition rate of tryptamine.

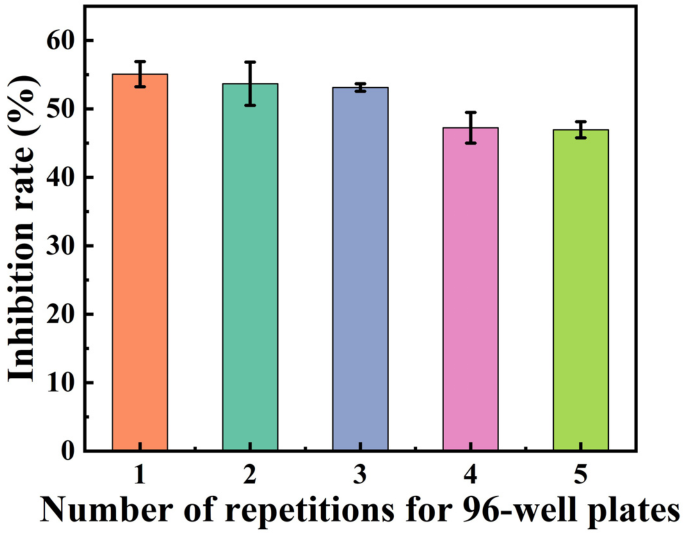

Figure S4. Resuability of the MIP-filled 96-well plate.

Table S1. Comparison of the Michaelis-Menten constant (K_m) and maximum reaction rate (V_{max}) for different catalysts.

Catalysts	K_m (mM)		V_{max} (M s^{-1})		Reference
	TMB	H_2O_2	TMB	H_2O_2	
**HRP**	0.43	3.70	10.00×10^{-8}	8.71×10^{-8}	[30]
CuO@Au NPs	21.56	236.46	17.89×10^{-6}	19.30×10^{-6}	This work

Table S2. Chemical structures, IC_{50} and cross-reactivity of histamine and its structural analogues.

**Analyte**	**Structure**	**IC_{50} (mg L^{-1})**	**CR (%)**
Histamine	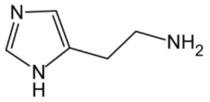	6.80	100.00
Phenethylamine	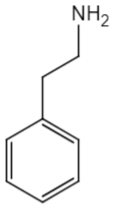	30.47	22.32
Tyramine	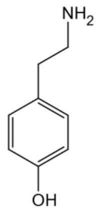	59.91	11.35

Table S3. Chemical structures, IC_{50} and cross-reactivity of tryptamine and its structural analogues.

Analyte	Structure	IC_{50} (mg L^{-1})	CR (%)
Tryptamine	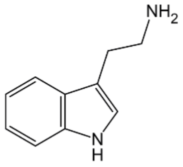	12.02	100.00
Phenethylamine	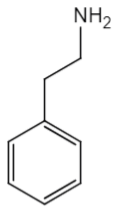	46.46	25.86
Tyramine	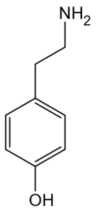	59.16	20.31

Table S4. Comparison of the BELISA method and HPLC method for the detection of histamine and tryptamine in different foods (n=3).

Samples	Analyte	Found level by BELISA (mg L^{-1})	Found level by HPLC (mg L^{-1})
Fish	Histamine	10.78 ± 0.42	10.39 ± 0.05
	Tryptamine	0.64 ± 0.02	0.67 ± 0.09
Soy sauce	Histamine	10.96 ± 0.66	11.05 ± 0.08
	Tryptamine	17.34 ± 0.48	16.75 ± 0.07
Rice vinegar	Histamine	4.88 ± 0.31	4.94 ± 0.08
	Tryptamine	7.49 ± 0.44	7.39 ± 0.19

Table S5. Comparison of the BELISA method with other methods for histamine and tryptamine detection.

Methods	Analyte	LOD (mg L^{-1})	Reference
Electrochemical immunosensor	Histamine	0.20	[26]
ELISA	Histamine	0.50	[27]
SERS	Tryptamine	0.07	[28]
LC-UV	Tryptamine	0.30	[29]
BELISA	Histamine	0.04	This work
	Tryptamine	0.14	
